# Supplementary material for: An Increased Risk of Stunting among Newborns in Poorer Rural Settings: A Cross-Sectional Pilot Study among Pregnant Women at Selected Sites in Rural Cambodia
Source: Int J Environ Res Public Health. 2019 Oct 29;16(21):4170. doi: 10.3390/ijerph16214170 (PMC6861886; doi:10.3390/ijerph16214170)
Supplement: Supplementary file 1 [file ijerph-16-04170-s001.pdf]

# The Questionnaires

The subject volunteered to participate in this study and agreed that we extract necessary information from their hospital records after delivery.

If you have questions or need more information, please do not hesitate to call

To …………………………………………………..

Subject ID: …………………….

Area Code: ……………………. Health Center: ……………………

**SECTION A: MATERNAL SOCIOECONOMIC INFORMATION**

1. Patients name: …………………. Age:…………….
2. Address: ……………Village…………………Commune…………District:……………….
   Telephone number:…………………
3. Gravida.....................Para:………………………
4. Education ………………………Occupation ………………………..
5. Husband? ❑ Death ❑ A live ➲ If alive how old is he?:……………
   ➲ Education........................... ➲ Occupation...............................
6. Major illnesses of mother, ❑ No ❑ Yes, Specify…………………………………
7. Medication of mother if daily, ❑ No ❑ Yes, Specify…………………………………
8. How many children do you already have?
   ➲ Baby Girl: ................................., Age……………………………………..
   ➲ Baby Boy: ................................. Age……………………………………..
   ➲ Stillborn?:……………………..
   ➲ Died under five?:…………………………..
9. Breastfeeding of previous babies, ❑ No ❑ Yes, if yes how long…………………
10. How long have you lived at present address: Years:…………………………….
11. How do you describe your area of residence? ❑ Urban ❑ Rural
12. Sources of drinking water? ❑ Tap ❑ Lake ❑ Rain water ❑ Borehole ❑ Bottle

Other, specify:………………………………………………………………………..

13. Do you use pesticides ❑ No ❑ Yes, if yes where? ❑ Inside home ❑ Rice Field ❑ On farm/garden
14. Do you or member of family fish? ❑ No ❑ Yes, if yes where?
    ❑ Lake ❑ Stream ❑ Large River

**SECTION B: DIET AND LIFESTYLE**
**Daily food sources of mother**

| Type of Food | Description | | | | Frequency | | | | | | | | |
|---|---|---|---|---|---|---|---|---|---|---|---|---|---|
| | Medium serving size | Small | Medium | Large | Never | 1/month | 2-3 month | 1/week | 2/week | 3-4/week | 5-6/week | 1/day | 2+/day |
| Meat | | | | | | | | | | | | | |
| Beef | 100g | | | | | | | | | | | | |
| Pork | 100g | | | | | | | | | | | | |
| Chicken | 200g | | | | | | | | | | | | |
| Fish | | | | | | | | | | | | | |
| Fresh Fish | 100g | | | | | | | | | | | | |
| Dry salty Fish | 50g | | | | | | | | | | | | |
| Smoke Fish | 50g | | | | | | | | | | | | |
| Vegetable | | | | | | | | | | | | | |
| Salad | 50g | | | | | | | | | | | | |
| Cabages | 100g | | | | | | | | | | | | |
| Spinages | 100g | | | | | | | | | | | | |

| | | | | | | | | | | | | | |
|---|---|---|---|---|---|---|---|---|---|---|---|---|---|
| water convolvulus | 50g | | | | | | | | | | | | |
| Cucomber | 50g | | | | | | | | | | | | |
| Eggplants | 50g | | | | | | | | | | | | |
| luffa gourds | 50g | | | | | | | | | | | | |
| Pumkins | 50g | | | | | | | | | | | | |
| Moringa | 50g | | | | | | | | | | | | |
| Beans | 50g | | | | | | | | | | | | |
| Bean Sprouse | 50g | | | | | | | | | | | | |
| Egg | 1 | | | | | | | | | | | | |
| Papaya | 100 | | | | | | | | | | | | |
| Others | | | | | | | | | | | | | |
| Rice | 100g | | | | | | | | | | | | |
| Soya Bean | 50g | | | | | | | | | | | | |
| Mung Beans | 5og | | | | | | | | | | | | |
| Sweat Potatoe | 1 | | | | | | | | | | | | |
| Sugar Can Juice | 1 cup | | | | | | | | | | | | |
| Corn | 1 | | | | | | | | | | | | |
| Coconut | 1 | | | | | | | | | | | | |
| Banana | 1 | | | | | | | | | | | | |

| | | | | | | | | | | | | | |
|---|---|---|---|---|---|---|---|---|---|---|---|---|---|
| Mango | 1 | | | | | | | | | | | | |
| Pinapple | 1 | | | | | | | | | | | | |
| Milk | 1 can | | | | | | | | | | | | |

15. Sources of food: ❑ Own/local production ❑ Local market
❑ Supermarket ❑ Imported

**Lifestyle of mother**

- Do you smoke? ❑ No
  ❑ Yes, ⇨If yes how many cigarettes daily…………………

- Do you drink alcohol
  ❑ No
  ❑ Yes, ⇨ If yes what kind……………………
  ⇨ How many tots daily…………………

**SECTON C: MATERNAL INFORMATION**
**POST DELIVERY MEDICAL INFORMATION**

To be completed by attending Health Center Staff or designated field worker.

1. Maternal weight before delivery: ….. Kilograms …………
2. Maternal height: ……………………cm……………………….
3. Previous spontaneous abortions 1. trimester: (if available)………..
   1 at how many months
   2 at how many months
   3 at how many months

4. Previous spontaneous abortions 2. trimester (if available)………
   1 at how many months
   2 at how many months
   3 at how many months
5. Previous preterm deliveries <week 37 (if available)……………
   1 at how many months
   2 at how many months
   3 at how many months
6. Any infertility problems –time to pregnancy – if any:
   ………………………………………………………………………
7. Any complication/problems during pregnancy (hypertension, pre-eclampsia, infections)…………………………………………………………………..

**SECTION D: INFORMATION ABOUT THE NEWBORN CHILD**

1. Mode of delivery: ❑ Nornal Deliver ❑ Vacum ❑ CS

2. Birth weight of baby: ………………kg…..
3. Birth length of baby: …………………cm
4. Head circumference of baby: …………cm
5. Gestation age of baby (based on Naegele term):
6. APGAR score 1min……… 5min………
   Any sign of asphyxia? ❑ No ❑ Yes
7. Gender of baby……………
8. Congenital malformations (visible at birth) ❑ No ❑ Yes
9. Any other medical observations or conditions
   ❑ No ❑ Yes, Specify .............................................……………………
